# Environmental Pollutants Effect on Brown Adipose Tissue

**DOI:** 10.3389/fphys.2018.01891

**Published:** 2019-01-09

**Authors:** Ilaria Di Gregorio, Rosa Anna Busiello, Mario Alberto Burgos Aceves, Marilena Lepretti, Gaetana Paolella, Lillà Lionetti

**Affiliations:** Department of Chemistry and Biology “A. Zambelli”, University of Salerno, Fisciano, Italy

**Keywords:** brown adipocytes, air pollutants, dichlorodiphenyltrichoroethane (DDT), dichlorodiphenylethylene (DDE), perfluorooctane sulfonate (PFOS), perfluorooctanoic acid (PFOA), obesity, metabolic disorders

## Abstract

Brown adipose tissue (BAT) with its thermogenic function due to the presence of the mitochondrial uncoupling protein 1 (UCP1), has been positively associated with improved resistance to obesity and metabolic diseases. During recent years, the potential influence of environmental pollutants on energetic homoeostasis and obesity development has drawn increased attention. The purpose of this review is to discuss how regulation of BAT function could be involved in the environmental pollutant effect on body energy metabolism. We mainly focused in reviewing studies on animal models, which provide a better insight into the cellular mechanisms involved in this effect on body energy metabolism. The current literature supports the hypothesis that some environmental pollutants, acting as endocrine disruptors (EDCs), such as dichlorodiphenyltrichoroethane (DDT) and its metabolite dichlorodiphenylethylene (DDE) as well as some, traffic pollutants, are associated with increased obesity risk, whereas some other chemicals, such as perfluorooctane sulfonate (PFOS) and perfluorooctanoic acid (PFOA), had a reverse association with obesity. Noteworthy, the EDCs associated with obesity and metabolic disorders impaired BAT mass and function. Perinatal exposure to DDT impaired BAT thermogenesis and substrate utilization, increasing susceptibility to metabolic syndrome. Ambient particulate air pollutions induced insulin resistance associated with BAT mitochondrial dysfunction. On the other hand, the environmental pollutants (PFOS/PFOA) elicited a reduction in body weight and adipose mass associated with upregulation of UCP1 and increased oxidative capacity in brown-fat mitochondria. Further research is needed to better understand the physiological role of BAT in response to exposure to both obesogenic and anti-obesogenic pollutants and to confirm the same role in humans.

## Introduction

The potential influence of environmental pollutants on energetic homoeostasis and obesity development by altering brown adipose tissue (BAT) thermogenic activity has recently drawn increased attention (reviewed in Zhang et al., 2016). Obesity is a condition that develops when energy intake is greater than energy expenditure and energy surplus is accumulated in white adipose tissue (WAT). Indeed, WAT, characterized by the presence of white unilocular adipocytes, is typically considered an energy storage site in condition of chronic positive energy balance, but it also contributes to energy balance regulation by releasing special adipokines (Trayhurn and Beattie, 2001; Vázquez-Vela et al., 2008). The role of adipose tissue as a potential site of toxicant accumulation has been recently reviewed (Jackson et al., 2017). On the other, BAT is characterized by brown multilocular adipocytes and is involved in the activation of thermogenesis due to the presence of the uncoupling protein 1 (UCP1), that may be useful for increasing energy expenditure (Ricquier, 2005; Richard, 2007; Marcelin and Chua, 2010; Busiello et al., 2015) and avoiding excessive lipid accumulation in WAT, counteracting obesity and metabolic diseases development (Cannon and Nedergaard, 2004; Nedergaard et al., 2007; Saito et al., 2009; Ouellet et al., 2011; Lombardi et al., 2015). Indeed, recent literature suggested BAT as a prime target for the treatment of obesity and metabolic diseases by regulating energy expenditure (Blondin and Carpentier, 2016; Scheele and Nielsen, 2017; Trayhurn, 2017; Carpentier et al., 2018). However, although the role of BAT on thermogenesis and energy metabolism, is well-established in rodents, the contribution of BAT to energy balance in humans is more controversial. A detailed discussion on this topic is beyond the role of this review and has been well-analyzed in recent reviews (please see Trayhurn, 2017; Carpentier et al., 2018). We briefly summarized the main findings that provide the rationale for BAT thermogenesis involvement in energy metabolism regulation and obesity in rodents as well as in humans. Brown adipose tissue (BAT) was first identified as the major site of non-shivering thermogenesis in rats acclimated to cold and this finding had impact also in the research area of nutritional energetic where thermogenesis was starting to be considered a significant factor in obesity onset (Trayhurn, 2017). The first demonstrations of a link between obesity and BAT were obtained in genetic obese mice (ob/ob mice), where a reduction in BAT thermogenic activities was found, as well as in cafeteria-fed rats where an activation of BAT was evident (Rothwell and Stock, 1979, 1981; Thurlby and Trayhurn, 1979). These initial observations were followed by several studies in other genetic and non-genetic models of obesity, confirming a link between BAT thermogenesis and obesity in rodents (see Trayhurn, 2017). A critical point is whether the data showing the link between BAT and obesity in animal experimental models, may suggest a similar correlation in humans. Studies in the 1980s provided a clear evidence that BAT not only is present in the newborns, but also in adult humans, showing the presence of UCP1 in particular adipose depots, and its activation in patients with phaeochromocytoma (Lean et al., 1986a,b). Two key discoveries in the late 2000s elicited a renaissance interest in BAT: (1) brown adipocytes, differently from white fat cells, are derived from myogenic precursors (Timmons et al., 2007) and (2) there is a third type of adipocyte (beige or brite fat cell) which express UCP1 together with other molecular markers of brown adipocyte (Petrovic et al., 2010; Wu et al., 2012). Moreover, flurodeoxyglucose positron emission tomography (FDG-PET) and UCP1 immunostaining studies showed that BAT activity is also present in adult humans (Cypess et al., 2009; Virtanen et al., 2009). Further FDG-PET studies showed that: (1) BAT in adults is stimulated by cold and insulin (van Marken Lichtenbelt et al., 2009; Orava et al., 2011), (2) it is less active in older subjects (Pfannenberg et al., 2010); (3) its activity inversely correlate with BMI (body mass index) and therefore lower in obese than in lean individuals (van Marken Lichtenbelt et al., 2009; Pfannenberg et al., 2010; Yoneshiro et al., 2011). Moreover, genetic variants of UCP1 are associated with fat metabolism, obesity, and diabetes (Jia et al., 2010). The finding that brown-fat like adipocytes are also present in WAT has opened new opportunities in targeting the adipose tissue to fight obesity-induced diseases (Cereijo et al., 2015; Bargut et al., 2017). Indeed, alteration in adipose tissue thermogenic gene expression has been found in response to obesity and diabetes (Keller and Attie, 2010; Marcelin and Chua, 2010; Ruschke et al., 2010). Moreover, it has been also suggested that WAT and BAT, in spite of their opposite functions, share the ability for reciprocal reversible transdifferentiation in response to physiologic needs. Thus, chronic positive energy balance has been suggested to induce whitening, whereas chronic need for thermogenesis has been suggested to induce browning (Cinti, 2011, 2018). Although there are some concerns on whether BAT facultative thermogenesis can be more than a very minor component of energy expenditure in adult humans, these last discoveries lead to a renewed interest in the activation and/or recruitment of BAT in the etiology and therapy of obesity and related metabolic diseases in humans.

Given the possible role played by BAT in the etiology of obesity, the purpose of this review is to summarize the current knowledge on the involvement of BAT in the effect of environmental pollutants on body energy metabolism by considering the opposite effect of obesogenic and anti-obesogenic pollutants. We focused in reviewing mainly rodent studies, either *in vitro* or *in vivo*, where the used methodologies allow to clarify cellular mechanisms involved in pollutants effect on BAT activity and obesity development. As regard obesogenic pollutants, we reviewed the literature on endocrine disruptors (EDCs), such as dichlorodiphenyltrichoroethane (DDT), and its primary metabolite dichlorodiphenyldichloroethylene (DDE) (Lee et al., 2011; Taylor et al., 2013; La Merrill et al., 2014) as well as on airborne fine particulate matter (traffic pollutants) (Xu et al., 2010, 2011a,b), which have been shown to impair BAT mass and function in association with the development of obesity and/or metabolic related diseases. Concerning anti-obesogenic pollutants„ we discussed the effect of perfluorooctane sulfonate (PFOS) and perfluorooctanoic acid (PFOA), which have a reverse association with obesity and are associated with UCP1 upregulation and increased oxidative capacity in brown-fat mitochondria (Shabalina et al., 2015, 2016). The opposite effects of these different environmental pollutants suggest that further studies are needed to clarify the metabolic response to xenobiotic agents. Moreover, targeting brown adipocyte and mitochondria could be useful in the prevention and therapy of obesity and metabolic related diseases.

## Environmental Pollutants Inducing Obesity and Metabolic Disorders: the Role of BAT

### Dichlorodiphenyltrichoroethane (DDT) and Dichlorodiphenylethylene (DDE) Impact on BAT Function

Exposure to DDT and its primary metabolite DDE has been associated with increased prevalence of obesity and metabolic disease (Lee et al., 2011; Taylor et al., 2013).

**Figure 1:**
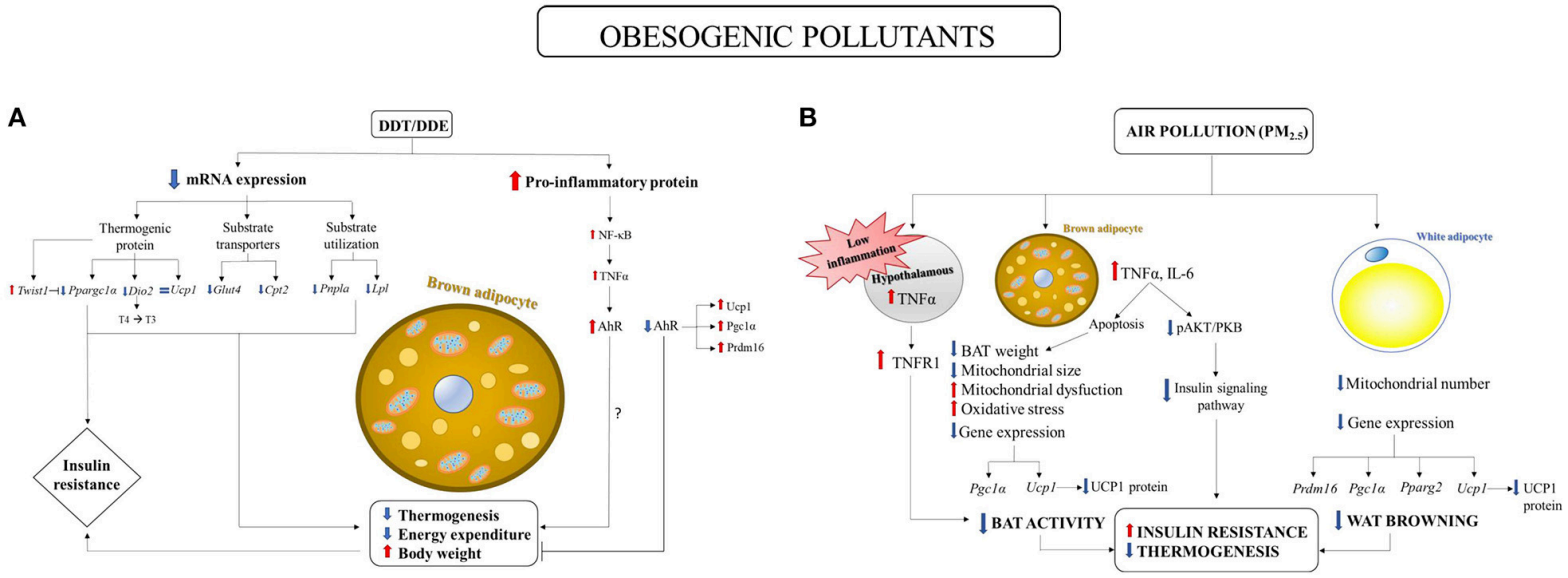
Environmental pollutant-induced obesity and metabolic diseases: involvement of BAT impairment. **(A)** Perinatal exposure to DDT in mice elicited reductions in mRNA expression of: (1) proteins involved in substrates transporters: glucose transporter type 4 (*Glut4*) and carnitine palmitoyltransferase 2 (*Cpt2*); (2) proteins involved in substrate utilization: adipose triglyceride lipase (*Pnpla*) and lipoprotein lipase (*Lpl*); (3) proteins involved in thermogenesis: iodothyronine type II deiodinase (*Dio2*, which encodes the enzyme that locally activates thyroid hormones through the deiodination of T4 to T3) and peroxisome proliferative activated receptor gamma coactivator 1 alpha (*Ppargc1a*, a master regulator in the thermogenesis), associated with an increase in the expression of twistrelated protein 1 (*Twist1*), a negative regulator of *Ppargc1a*. (La Merrill et al., 2014). *Ppargc1a* reduction may induce insulin resistance (Kleiner et al., 2012). No variation was found in Ucp1 mRNA expression (La Merrill et al., 2014). Aryl hydrocarbon receptor (AhR) deficiency induced increases in BAT thermogenic genes expressions: UCP1, PPARγ coactivator 1 α (PGC1α) and PR domain containing 16 (PRDM16) (Xu et al., 2015). DDT/DDE may have pro-inflammatory effect by inducing NF-κB activation and tumor necrosis factor α (TNFα) production (Kim et al., 2004) which in turn may activate aryl hydrocarbon receptor (AhR) signaling pathway. **(B)** Long term exposure to PM_2.5_ has the following impact on BAT: (1) decreased BAT weight and mitochondrial size; (2) increased mitochondrial dysfunction and oxidative stress; (3) decreased *Ucp1* and *Pgc-1*α gene expression and Ucp1 protein content. The effects on WAT were: (1) decreased mitochondrial number; (2) decreased *Ucp1, Prdm16, Pgc-1*α, and *Pparg2* gene expression and UCP1 protein content. (Xu et al., 2011b). PM_2.5_ is associated with BAT inflammation through the increased expression of interleukin-6 (IL6) and tumor necrosis factor α (TNFα), which negatively regulate BAT development by inducing apoptosis (Porras et al., 1997; Valladares et al., 2000; Liu et al., 2014). BAT inflammation was associated to decrease in ser437 phosphorylation of AKT and the impairment of insulin signaling in BAT (Xu et al., 2011a). PM_2.5_ also induced low increase in hypothalamic TNFα concentration which lead to the activation only of TNF receptor 1 (TNFR1), which has opposite effect to TNFR2 by decreasing BAT function (Chen and Palmer, 2013).

Perinatal exposure to DDT in mice has been proved to decrease energy expenditure leading to increased body weight and insulin resistance, associated with BAT activity impairment in female adult offspring (La Merrill et al., 2014). In fact, reductions in the expression of numerous RNA involved in thermogenesis and substrate transport and utilization were found in BAT of female adult offspring fed high fat diet (see Figure 1A). Noteworthy, the authors also showed a reduction in iodothyronine type II deiodinase (*Dio2*). *Dio2* encodes the enzyme that locally activates thyroid hormones through the deiodination of T4 to T3, therefore stimulating BAT thermogenesis. La Merrill et al. (2014) also established a link between developmental exposure to DDT and increased risk of insulin resistance in adult offspring. It should be noted that peroxisome proliferative activated receptor gamma coactivator 1 alpha *(*PGC1α*)* has been suggested to play a role in linking thermogenesis to the risk of T2D. Indeed, thermogenesis impairment and HFD-induced insulin resistance susceptibility were observed in *Ppargc1a* genetic ablation studies in mice (Kleiner et al., 2012). La Merrill et al. (2014) therefore suggested a role for PGC1α reduction in thermogenesis and insulin resistance in DDT exposure adult offspring (Figure 1A).

The effect of DDT/DDE and other obesogenic xenobiotics on BAT may be mediated by the aryl hydrocarbon receptor (AhR) (Xu et al., 2015). AhR, initially characterized as a xenobiotic sensor to mediate toxicological response, has been also shown to act as a physiological regulator of energy metabolism. Therefore, AhR activation by environmental pollutants has been suggested to promote obesity and related diseases (Arsenescu et al., 2008; He et al., 2013). Indeed, AhR deficiency in mice protected against diet-induced obesity and metabolic disorders by stimulating energy expenditure through increased BAT activity (Figure 1A) (Xu et al., 2015). UCP1 and the key upstream regulators of Ucp1, PPARγ coactivator 1 α (PGC1α) and PR domain containing 16 (PRDM16), were increased in the BAT of AhR^+/−^ mice compared to control mice (Xu et al., 2015). Taking into account the role of inflammatory pathways in obesity and related diseases, it is important to underline that AhR activation has been shown to be enhanced by pro-inflammatory cytokines (Champion et al., 2013; Drozdzik et al., 2014) and to play a key role in inflammatory pathways. Considering that DDT and its metabolite DDE have been shown to induce NF-κB activation and pro-inflammatory cytokines production (Kim et al., 2004; Alegría-Torres et al., 2009), which in turn may mediate the upregulation of AhR expression, the role of AhR over-expression induced by xenobiotics in BAT as well as its link with the inflammation, deserve further investigation.

### Air Pollutants Impact on BAT Function

Several studies showed a link between air pollution and obesity and metabolic disorders development (Araujo et al., 2008; Sun et al., 2009; Xu et al., 2010). Xu et al. (2011a) showed that long term exposure to airborne fine particulate matter (PM <2.5 μm in aerodynamic diameter, PM_2.5_) induced insulin resistance and inflammation, associated with a reduction in BAT weight, a significant decreased mitochondrial size in BAT and in mitochondrial number in WAT. Moreover, brown adipocyte-specific gene profiles and UCP1 content were impaired in both BAT and WAT, suggesting that long-term PM_2.5_ exposure induces alterations in BAT activity and in browning of WAT (Figure 1B). These findings were associated with impaired glucose tolerance, insulin resistance and inflammation.

Evidence of significant changes in BAT activity in response to PM_2.5_ was also found in male APOE knockout mice (Xu et al., 2011b). PM_2.5_ exposure elicited a significant downregulation of brown adipocyte-specific gene profiles as well as a decrease in UCP1 content and in mitochondrial size and number in BAT. Noteworthy, homeobox C9 (*Hoxc9*) and insulin-like growth factor binding protein 3 (*Igfbp3*) genes, which are characteristic of WAT, were upregulated in BAT, suggesting that brown adipocyte may potentially transform to white adipose phenotype_._ This suggestion opens an interesting perspective in the studies on BAT involvement in toxicant-induced obesity and in the potential shift of BAT in WAT, in accordance with the hypothesis that brown to white conversion can be useful to meet the need of storing excess energy in response to obesogenic stimuli, as previously discussed (Cinti, 2011, 2018).

The effects of PM_2.5_ exposure on BAT activity was also confirmed by the finding of the induction of BAT mitochondrial dysfunction and oxidative stress in both PM_2.5_ long term-exposed mice and PM_2.5_ exposed APOE knockout mice (Xu et al., 2011a,b). Zhang et al. (2016) also reviewed the hypothesis that exposure to PM_2.5_ may impact BAT development through TNFα mediated apoptosis (Porras et al., 1997; Valladares et al., 2000), inflammation (Liu et al., 2014) and insulin signaling pathway impairment (Figure 1A). PM_2.5_ is associated with systemic proinflammatory response in animals and humans (Calderon-Garciduenas et al., 2008; Xu et al., 2010), as well as with BAT inflammation through the increased expression of interleukin-6 (IL6) and TNFα (Liu et al., 2014). It has been shown that TNFα negatively regulates BAT development by inducing apoptosis (Porras et al., 1997; Valladares et al., 2000). Therefore, it has been suggested that exposure to PM_2.5_ may impact BAT development through TNFα mediated apoptosis and inflammation (Zhang et al., 2016). Brown adipose tissue (BAT) inflammation was associated to the impairment of insulin signaling pathway as demonstrated by the decrease in ser437 phosphorylation of AKT in BAT (Xu et al., 2011a). Moreover, PM_2.5_ exposure induced hypothalamic inflammation (Ying et al., 2014), which in turn may have a dual effect on BAT function. High concentration of hypothalamic TNFα increased BAT function by increasing UCP1 expression through sympathetic tonus activation (Arruda et al., 2010). On the other hand, low concentration of hypothalamic TNFα only activated tumor necrosis factor-alpha receptor 1 (TNFR1), which has an opposite effect to TNFR2 (Chen and Palmer, 2013) and decreased BAT thermogenic activity by downregulating UCP1 expression (Romanatto et al., 2009; Arruda et al., 2011). Exposure to PM_2.5_ has been suggested to induce low-grade inflammation in the hypothalamus, which in turn may induce BAT dysfunction (Zhang et al., 2016); Figure 1B.

PM_2.5_ exposure also induced increased expression of *TNF*α gene expression associated with decreased expression of BAT specific gene expression (*UCP1* and *PGC1*α) in epicardial adipose tissue (EAT) (Sun et al., 2013). These findings suggested that further investigations are needed in clarifying EAT function and air pollution effect on it.

### Phthalates and Bisphenol a Impact on BAT Function

The impact of other obesogenic environmental pollutants on BAT activity also needs to be further investigated. The endocrine disruptor mono-(2-ethylhexyl) phthalate (MEHP), a metabolite of the widespread plasticizer DEHP [di-(2-ethylhexyl) phthalate], promotes adipocyte differentiation and induces obesity in mice (Feige et al., 2010). In recent years, it has been shown that phthalates may promote childhood obesity (Kim and Park, 2014). However, BAT activity does not seem to be altered by phthalates, given that BAT UCP1 and PGC-1α expressions were not affected in DEHP-treated mice (Feige et al., 2010).

Epidemiologic evidence on the correlation between bisphenol A (BPA) exposure and obesity, diabetes, and other metabolic diseases has been reviewed by Bertoli et al. (2015). Nunez et al. (2001), showed that there was a preferential concentration of BPA in BAT in ovariectomized adult female rat. The authors therefore suggested that BAT may be a primary tissue into which BPA accumulates in mammals and, therefore, BPA can affect BAT thermogenesis and energy balance.

Further studies are needed to clarify the effect of phthalates and BPA on BAT and energy balance.

## BAT Role in Anti-Obesogenic Effect of Environmental Pollutants

The anti-obesogenic effect of PFOA and PFOS associated with increased BAT activity has been well-studied by the group of Nedergaard (Shabalina et al., 2015, 2016). PFOA/PFOS supplementation to the food elicited a marked decrease in body weight and WAT depots in mice (Xie et al., 2002, 2003), which has been shown to be due to the increased oxidative capacity in brown-fat mitochondria by UCP1 upregulation (Shabalina et al., 2015, 2016).

**Figure 2:**
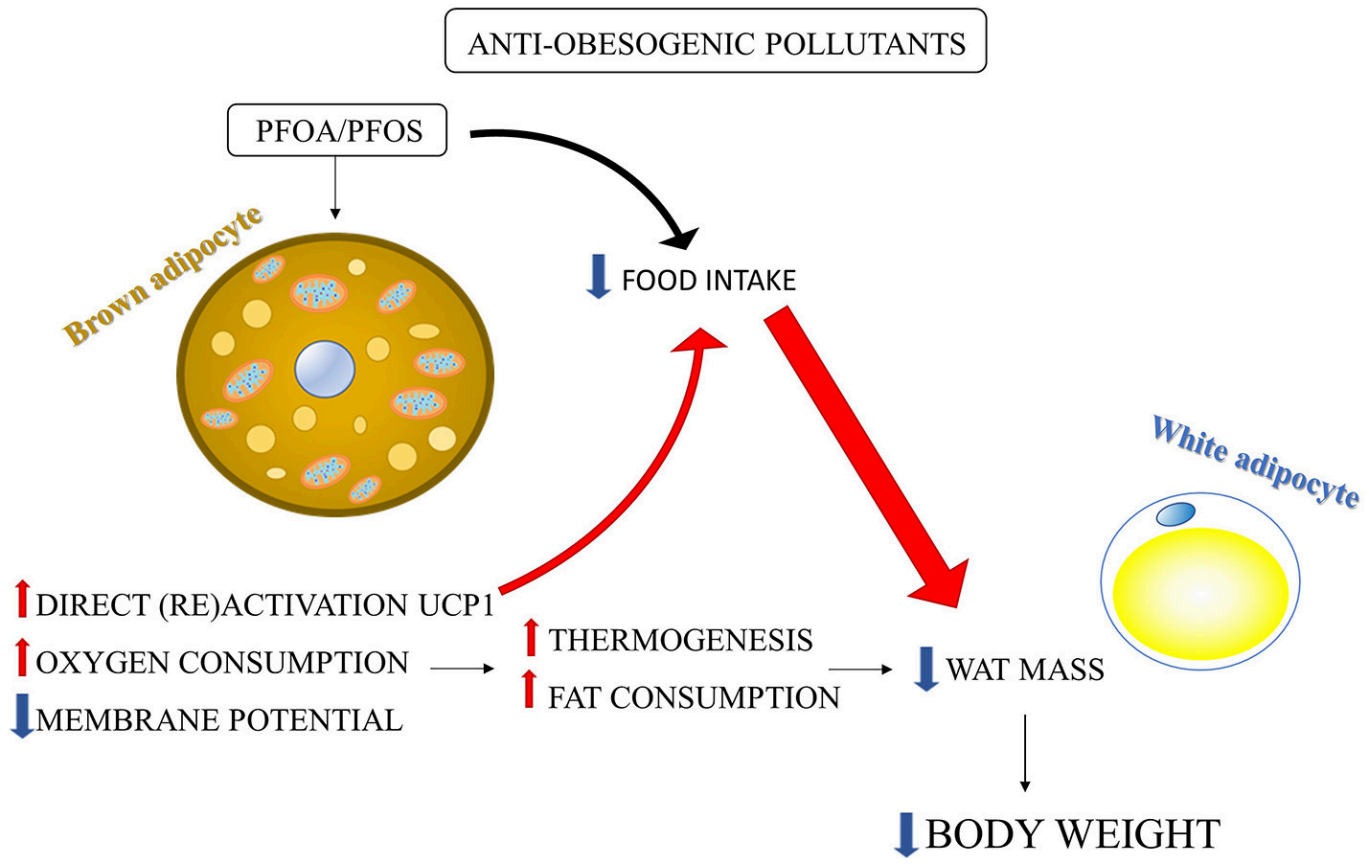
Environmental pollutants-induced body weight reduction: involvement of BAT activation. PFOS and PFOA induced in isolated brown-fat mitochondria: (1) direct (re) activation of UCP1; (2) mitochondrial membrane potential dissipation; (3) mitochondrial oxygen consumption increase. The consequent increase in fat combustion and energy expenditure may induce reduction in WAT mass and body weight. However, PFOS/PFOA-induced body weight reduction was mainly due to a reduction in food intake rather to the increase in thermogenesis. The large part of the PFOS/PFOS-induced food intake reduction was dependent on the presence of UCP1 in BAT (Shabalina et al., 2015, 2016).

PFOA and PFOS, with their fatty acid octanoic acid structure, are structurally similar to a fatty acid and may exhibit properties akin to those of natural fatty acids, such as their ability to (re)activate UCP1 (Cannon and Nedergaard, 2004). The activation of UCP1 leads to increased fat combustion and may explain, at least in part, the body fat loss induced by PFOA and PFOS (Figure 2), which may be also explained by an UCP1-dependent decrease in food intake (Shabalina et al., 2015, 2016). Shabalina et al. (2016) showed that PFOS and PFOA induced oxygen consumption and dissipated membrane potential by directly activating UCP1 in isolated brown-fat mitochondria. The direct activation of UCP1 was confirmed by the absence of these effects in brown-fat mitochondria from UCP1-ablated mice (Shabalina et al., 2016). Considering that BAT has been recently found also in adult human and its (re)activation may be useful to counteract obesity (Nedergaard et al., 2007; Cereijo et al., 2015), the finding that PFCs directly activates BAT can be useful to enable the development of substances that selectively activate UCP1 allowing recruitment of BAT to ameliorate obesity problem (Shabalina et al., 2016). Shabalina et al. (2015) also demonstrated that the PFOA/PFOS-induced body weight reduction was only in part directly related to the presence of UCP1. Indeed, it was mainly due to a decrease in food intake which in turn was dependent on the presence of UCP1 in BAT (Figure 2). Therefore, the reduction in body weight induced by PFOA/PFOS may be ascribed to a UCP1-dependent decrease in food intake (not due to food adversion), rather than to elevated BAT thermogenesis (Shabalina et al., 2015). A regulatory component of food intake depending upon BAT thermogenic activity can be suggested and further studies are needed to clarify this mechanism considering its possible key role in counteracting obesity.

## Conclusions

Diverse type of environmental pollutants differently affects body energy metabolism and BAT function. Among obesogenic pollutants, DDT/DDE impaired BAT activity by reducing the expression of protein involved in thermogenesis, substrate transport and utilization as well as by reducing the deiodination of T4 to T3 and inducing insulin resistance and inflammatory pathways in BAT. Air pollutants also induced inflammatory pathway and insulin resistance in BAT. Noteworthy, obesogenic effect of air pollutants was associated with downregulation of brown adipocyte-specific gene profiles as well as with a decrease in UCP1 content and mitochondrial size/number in both BAT and WAT, suggesting some common effects of environmental pollutants on adipose tissue in general. On the other hand, anti-obesogenic effect of PFOA/PFOS has been shown to be due to increased mitochondrial oxidative capacity and UCP1 activation in BAT, which in turn induced a decrease in food intake and body weight.

The different effects of diverse type of environmental pollutant may be due to the diverse chemical structure and proprieties as well as to the different dose and time of exposure utilized in the diverse experimental designs. Understanding the mechanisms by which obesogenic environmental pollutants impact BAT thermogenesis activity and body energy metabolism may be useful to shade light on the etiopathogenesis of obesity whereas the studies on the environmental pollutants acting impairing BAT function and reducing body weight may be useful in discovering new molecules to counteract obesity by directly targeting thermogenic adipocyte. Given that mitochondria play a key role in BAT and browned WAT thermogenesis, studies on mitochondrial bioenergetics are confirmed to be of great importance in understanding the etiopatogenesis of diet-induced (Putti et al., 2015a,b; Lepretti et al., 2018), and toxicant-induced (Llobet et al., 2015; Burgos-Aceves et al., 2018) obesity as well as in discovering new therapy for obesity related diseases.

## Author Contributions

ID and RAB equally contributed to the work, conducted bibliographic research, and wrote the initial draft. MABA conducted bibliographic research and revised the manuscript. ML and GP actively discussed and revised the manuscript. LL conceived the idea and wrote the final manuscript. All authors read and approved the final manuscript.

### Conflict of Interest Statement

The authors declare that the research was conducted in the absence of any commercial or financial relationships that could be construed as a potential conflict of interest.
